# Copulatory Dyskinesia: Pathognomonic Manifestation of Tardive Dyskinesia

**DOI:** 10.5334/tohm.585

**Published:** 2020-12-16

**Authors:** Vibhash D. Sharma, Harsh V. Gupta, Alberto J. Espay

**Affiliations:** 1 Department of Neurology, University of Kansas Medical Center, Kansas City, KS, US; 2 James J and Joan A Gardner Center for Parkinson’s Disease and Movement Disorders, Department of Neurology, University of Cincinnati, OH, US

**Keywords:** tardive dyskinesia, pelvic thrusting, copulatory dyskinesia, drug-induced movement disorders

## Abstract

**Background:**

Copulatory or pelvic thrusting dyskinesia is a subtype of tardive dyskinesia (TD) which is caused by exposure to dopamine blocking agents.

**Phenomenology shown:**

A man exhibiting rhythmic, stereotypical pelvic thrusting movements.

**Educational value:**

Recognition of copulatory dyskinesia as a distinctive iatrogenic disorder helps prevent unnecessary investigations and guides the implementation of corrective strategies.

Tardive dyskinesia (TD) is an iatrogenic movement disorder resulting from exposure to neuroleptics, also known as dopamine-receptor blocking agents (DRBAs). The disorder is characterized by repetitive, stereotypic movements involving various body regions including mouth, tongue, face, trunk and extremities. Unlike most iatrogenic disorders, in most patients the abnormal movements may persist or worsen after discontinuation of the offending agent [1]. We document the phenomenon of copulatory dyskinesia as a distinctive tardive phenotype.

A 71-year-old man with history of depression was treated with aripiprazole. After 9 months of treatment, slow gait, hypomimia and hypophonia prompted a switch to brexpiprazole, which modestly attenuated the parkinsonism but within a few months elicited slight pelvic movements without alleviating his parkinsonism. Brexpiprazole was discontinued after 3–4 months and within weeks of discontinuation there was nearly complete resolution of the parkinsonian features but the rhythmic pelvic movements, which he could only transiently suppress, became troublesome (Video 1). Clonazepam and propranolol yielded no benefits. Valbenazine lessened these movements but aggravated his parkinsonism. The abnormal movements have persisted for over 2 years despite brexpiprazole discontinuation.

**Video 1:** **Copulatory dyskinesia.** Rhythmic, stereotypical pelvic thrusting movements characteristic of this iatrogenic disorder. The movements worsen when performing motor tasks (finger tapping) as is typical of other forms of TD.

Copulatory or pelvic-thrusting dyskinesia is a unique subtype of TD complicating treatment with DRBAs. The slow rhythmicity of this patient’s dyskinesia, suggesting a stereotypy, distinguishes it from other disorders associated with “pelvic-thrusting”, such as psychogenic non-epileptic seizures and anti-N-methyl D-aspartate (NMDA) receptor encephalitis, in which there is variability of amplitude, frequency, and topographical distribution. Brexpiprazole, a third-generation antipsychotic, is a partial agonist at 5-HT_1A_ and D2 receptors and antagonist at 5-HT_2A_ receptors [2] and has not been previously reported to induce TD.

Besides copulatory dyskinesia, neuroleptic exposure can also yield other axial dyskinesias, including respiratory dyskinesia and Belly dancer’s dyskinesia. Respiratory dyskinesia manifests as irregular breathing, tachypnea and abnormal chest movements due to involvement of respiratory muscles [3]. Belly dancer’s dyskinesia or moving-umbilicus syndrome is characterized by writhing movements and contraction of the abdominal muscles, which tend to be difficult to suppress voluntarily [4].

A single-center (not yet replicated) retrospective study suggested that copulatory dyskinesia may more commonly be a complication of the antiemetic metoclopramide [5]. While there are no recommendations regarding treatment of copulatory dyskinesia, its management is similar to other forms of TD, including discontinuation of the offending agent and treatment with VMAT-2 inhibitors, such as tetrabenazine or newer analogues. As emphasized by this case, rapid discontinuation of the neuroleptic may worsen or cause withdrawal emergent dyskinesia [1,6]; a slow taper may reduce the odds of such withdrawal effect. Clonazepam and propranolol may be considered second-line treatments for TD [6].

## Disclosures

Drs. Sharma & Gupta report no disclosures. Dr. Espay has received grant support from the NIH and the Michael J Fox Foundation; personal compensation as a consultant/scientific advisory board member for Abbvie, Neuroderm, Neurocrine, Amneal, Acadia, Acorda, Kyowa Kirin, Sunovion, Lundbeck, and USWorldMeds; publishing royalties from Lippincott Williams & Wilkins, Cambridge University Press, and Springer; and honoraria from USWorldMeds, Acadia, and Sunovion.
